# Implementation of post-intensive care outpatient clinic (I-POINT) for critically ill COVID-19 survivors

**DOI:** 10.3906/sag-2106-306

**Published:** 2021-12-17

**Authors:** Burçin HALAÇLI, Arzu TOPELİ

**Affiliations:** 1 Division of Intensive Care Medicine, Department of Internal Medicine, Hacettepe University Faculty of Medicine, Ankara Turkey; 2 Member of COVID-19 Scientific Advisory Board of Turkish Ministry of Health

**Keywords:** SARS-CoV-2, long COVID, pandemic, acute respiratory distress syndrome, postintensive care syndrome, outcome

## Abstract

Although we have enough and cumulative information about acute effects of COVID-19, our knowledge is extremely limited about long-term consequences of COVID-19, in terms of its impacts and burdens on patients, families, and the health system. Considering the underlying pathophysiological mechanisms affecting all of the organ systems in critically ill COVID-19 patients who are admitted to intensive care units, the development of post-intensive care syndrome is inevitable. This situation brings along the development of long-COVID. These patients should be followed regarding cognitive, physical, and psychiatric aspects and necessary specialist referrals should be carried out. In this article, we are presenting the experience and recommendations of our center, as a guide for the establishment process of post-intensive care outpatient clinics for the critically ill patients who required intensive care admission due to COVID-19 and could be discharged.

## 1. Introduction

**Figure 1:**
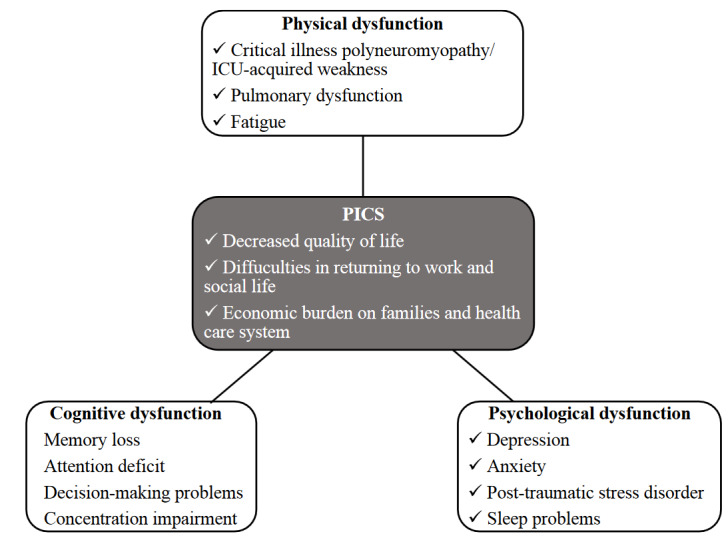
The domains of post-intensive care syndrome (PICS).

Coronavirus disease 2019 (COVID-19), a more severe viral respiratory failure agent than flu, causes hospitalization in 20% of the patients and intensive care unit (ICU) admission in an average of 5%–10% of the patients. Severe disease may present with severe acute respiratory infection (SARI), i.e. severe pneumonia and acute respiratory distress syndrome (ARDS) which is reported in 60%–70% of patients; sepsis and septic shock reported in 30%; myocarditis, arrhythmia, and cardiogenic shock in 20%–30%; and acute kidney injury in 10%–30% of critically ill COVID-19 patients [1]. ICU length of stay (LOS) of critically ill COVID-19 patients is quite long probably due to requirement of prolonged mechanical ventilation (>5–7 days), prolonged administration of corticosteroids, sedoanalgesia, and neuromuscular agents [2]. Even those with mild to moderate disease may have long-lasting symptoms up to 6 months [3]. With the improvement in the contemporary intensive care medicine, more patients can be discharged from ICUs. Approximately two-thirds of the ICU survivors are confronted with physical problems such as myo-polyneuropathy, ongoing dyspnea, cognitive and psycho-social problems such as anxiety, depression, posttraumatic distress, poor quality of life (QOL) [4,5]. This period is called “post-intensive care syndrome” (PICS), which can also have consequences for the families (PICS-Family) and caregivers of patients, and even brings an ongoing economic burden for the healthcare system [6]. The main domains of PICS are physical, cognitive and psychological dysfunctions which might be of concern after recovery and discharge to home (Figure 1).

ICU-acquired weakness (ICU-AW), which involves critical illness polyneuromyopathy including diaphragm weakness is reported to occur in about 43% of patients [7]. Main risk factors for the development of ICU-AW are prolonged mechanical ventilation, presence of sepsis and multiorgan failure, use of corticosteroids, aminoglycosides and neuromuscular blocker agents, prolonged heavy sedation, and hyperglycemia [8]. In addition, patients with ARDS lose 18% of their weight at ICU discharge and return to baseline weight occurs in only 71% of patients [9]. All of these factors cause prolonged mechanical ventilation, prolonged length of stay and therefore complications and even mortality. Physical functional impairment lasts for up to 5 years in ARDS survivors. At 5 years, the 6-minute walk distance was still lower than the predicted value regarding age and sex and physical component of Short Form Health Survey-36 (SF-36) used for QOL assessment was found to be impaired in ARDS survivors [10]. Moreover, the Bringing to Light the Risk Factors and Incidence of Neuropsychological Dysfunction in ICU Survivors (BRAIN-ICU) study [11] revealed depression in 30% of patients and post-traumatic stress disorder (PTSD) in 7% of ICU survivors for up to 1-year follow-up. QOL of ARDS survivors were still poor 1 year after discharge, as well [12].

It is often difficult for patients who are not physically competent to reach the hospital, so their complaints should be resolved and directed on time. Therefore, establishment of outpatient clinics for PICS has been suggested to manage these problems. These clinics were first founded in United Kingdom (UK) at the beginning of 1980s. It was recommended to evaluate the physical, cognitive functions, mental health, nutritional status, and QOL of patients in outpatient clinics for PICS, and to provide medical support accordingly. It was thought that the ongoing health problems of the patients could be eliminated and the burnout of patients and their relatives could be reduced [13]. There are many PICS outpatient programs varying from center to center; therefore, there is no optimal model. In some centers, only nurses follow the patients; however, in some centers physicians do follow patients one-to-one. There were several proposed and utilized outcome measurement tools for PICS which are summarized in Table. 

**Table:** Some of the assessment tools for post-intensive care syndrome (PICS) screening.

Parameters	Tools
Performance status	· Eastern Cooperative Oncology Group (ECOG)· Clinical Frailty Scale (CFS ©)· Modified Rankin Scale· Barthel Scale/Index
Pain status	· Visual analogue scale (VAS)
Dyspnea status	· Borg scale · Modified Medical Research Council dyspnea scale (mMRC)
Physical function	· Medical Research Council (MRC)· Hand dynamometry· 6-min walking test
Pulmonary function	· 6-min walking test· Spirometry· Maximum inspiratory and expiratory pressure (MIP/MEP)· Diffusion capacity of carbon monoxide (DLCO)
Nutritional status	· Nutritional Risk Screening (NRS)-2002· Malnutrition Universal Screening Tool (MUST)· Mini Nutritional Assessment Short Form (MNA-SF)
Cognitive function	· Mini-mental test (MMT)· Montreal Cognitive Assessment (MoCA)
Mental health	· Hospital Anxiety and Depression Scale (HADS)· Post-traumatic Stress Disorder-Diagnostic and Statistical Manual of Mental Disorders-5 Questionnaire(PTSD-DSM-5)· Impact of Events-Revised (IES-R)
Quality of life	· European quality of life questionnaire-5 dimensions 3/5 Level Versions (EQ-5D-3L/5L)· Short Form Health Survey-12 (SF-12)· Short Form Health Survey-36 (SF-36)

## 2. Long-COVID 

Previous experiences gained from severe acute respiratory syndrome (SARS) and Middle East respiratory syndrome (MERS) outbreaks that developed secondary to the coronavirus displayed that persistent symptoms and prolonged consequences were occasionally confronted. As a matter of fact, the prolonged effects of severe COVID-19, which has the potential to affect almost all organ systems, mainly the respiratory system, are inevitable. It was postulated that postacute period could be separated into two parts as subacute/ongoing COVID-19 seen in 4–12 weeks and chronic/post-COVID-19 beyond 12 weeks [14]. These time periods were somewhat arbitrarily defined. Although these definitions were proposed for this situation, the “long-COVID” Rajan S, Khunti K, Alwan N et al. In the wake of the pandemic: Preparing for Long COVID. Copenhagen (Denmark): European Observatory on Health Systems and Policies; 2021. (Policy Brief, No. 39.) [online]. Website: https://www.ncbi.nlm.nih.gov/books/NBK569598/ [accessed February 2021]. terminology has generally been accepted [15]. Long-COVID was described as “signs and symptoms that develop during or following an infection consistent with COVID-19 and which continue for more than 4 weeks and are not explained by an alternative diagnosis according to the National Institute for Health and Care Excellence (NICE) guideline NICE. Covid-19 rapid guideline: managing the long-term effects of covid-19 [online]. Website: https://www.nice.org.uk/guidance/ng188/chapter/4-Planning-care [accessed December 2020].. 

Potential pathophysiological explanations of long-COVID were virus-related impacts, immunological damage subsequent to the acute viral infection and occurrence of chronic critical illness due to COVID-19 [14]. It is estimated that about 10% of patients with COVID-19 experience long-COVID [16]. 

According to a recent study [16] searching attributes and predictors of long-COVID, it was characterized by symptoms of fatigue, headache, dyspnea, and anosmia and was more likely associated with increased age and body mass index, and female sex. They revealed that experiencing more than five symptoms during the first week of COVID-19 increased the probability of development of long-COVID by 3.5 times. A propensity matched cohort study [17] matched by age, sex, race, ethnicity, clinical conditions, urbanicity, region and month of hospitalization with non-COVID patients using inpatient and outpatient data showed that late health consequences in 1–4 months following COVID-19 diagnosis could directly be imputed to COVID-19. Of patients, 7.0% and 7.7% experienced post-COVID conditions during 1st- and 4th-month controls, respectively, after the initial COVID-19 inpatient hospitalization. COVID-19 patients had 2.8 times increased acute pulmonary embolism event compared to matched control. They also declared more nonspecific chest pain, fatigue, headache, and respiratory, nervous, circulatory, and gastrointestinal system symptoms than the control group. 

Another study [18] from the United States Department of Veterans Affairs electronic database, comparing COVID-19 patients who survived but did not require hospitalization with non-COVID outpatients demonstrated that patients with COVID-19 had increased risk of mortality (hazard ratio:1.59), respiratory system sequalae and neurocognitive problems, nervous system, mental health, metabolic, cardiovascular, and gastrointestinal disorders after the 1st month of illness. The predicted excess death was 8.39 per 1000 patients and there was increased need of outpatient support (hazard ratio:1.20) in COVID-19 patients at 6 months. They also showed increased use of several therapeutics including pain medications (opioids and nonopioids), antidepressants and anxiolytics. It seems that patients with long-COVID have an extensive burden on the health system, even in primary care [19]. 

Critically ill COVID-19 patients surviving intensive care are at an extremely high risk of developing PICS. COVID-19 may cause sequelae in almost all organ systems after the acute critical illness. Pulmonary fibrosis and bronchiectasis are commonly observed. In a single-center prospective, longitudinal study [20] in severe COVID-19 patients (n = 83) who did not require mechanical ventilation, during the control visits at 3, 6, 9, and 12 months, patients were tested for diffusing capacity of the lungs for carbon monoxide (DLCO), forced expiratory flow between 25% and 75% of forced vital capacity (FVC); functional residual capacity, and 6-min walking test (6-MWT), as well as dyspnea assessment using a modified Medical Research Council scale (mMRC). They found a significant decline in DLCO over the study period, with a median of 77% of predicted at 3 months, 76% of predicted at 6 months, and 88% of predicted at 12 months after discharge. A multicenter cohort study [21] consisting 113 COVID-19 survivors also revealed impaired DLCO percent predicted, reduced 6-min walk distance and exercise-induced oxygen desaturation. DLCO percent predicted was found to be the robust independent predictor related with severe and critical disease. Therefore, comprehensive assessments and planning are needed for the follow-up of COVID-19 patients. 

## 3. Post-intensive care follow-up of COVID-19 patients

Several methods and programs have been proposed to monitor patients recovering from COVID-19 in terms of PICS. Due to the extremely complex multiorgan support treatment necessity of critically ill COVID-19 patients during the disease process, post-COVID follow-up, and rehabilitation should also be planned in multiple layers. However, due to the nature of COVID-19 as a contagious disease, the lockdown practices that have been initiated and the exhausted ICU survivors who are unable to physically visit follow-up clinics, some telemedicine methods were recommended, as well [22]. A telephone screening tool named COVID-19 Yorkshire Rehabilitation Screening (C19-YRS) tool has been developed in UK by multidisciplinary-rehabilitation teams consisting of physiotherapists, occupational therapists, speech and language therapists, psychologists, dietitians and physicians in rehabilitation medicine, specialists from respiratory medicine and intensive care medicine [23]. They used virtual meeting methods to screen a list of potential long-term problems including breathlessness, voice, swallowing, nutrition, mobility, fatigue, personal care, usual activities, pain/discomfort, anxiety, depression, PTSD, continence, and cognition [24]. It has been recently inaugurated a randomized controlled trial with an ICU-specific virtual reality intervention on treatment of psychological disorders and QOL after COVID-19 [25]. Virtual reality is a relatively new technique that has been proven to be effective for treating several psychological impairments by supporting possible delusional memories which mainly cause psychological distress [26]. 

However, it is emphasized that this kind of multidisciplinary patient follow-up cannot be settled down only remotely. Therefore, some follow-up models that integrate both remote and face-to-face methods have been suggested. In one proposal from the UK [27], COVID-19 patients were assessed with virtual clinic by a multidisciplinary team 8–12 weeks after discharge. Noncritical patients were directed to general practitioners. Critically ill COVID-19 patients were evaluated in person 12 weeks after discharge. If patients have abnormal chest X-ray or respiratory physiology, they were referred to respiratory specialty clinic. If patients were interpreted as having significant functional impairment, they were followed in COVID survivorship clinic up to 6 and 12 months. A similar hybrid program from the United States, named Comprehensive Post-COVID Center at Yale (RECOVERY) recommends regular controls for a minimum of 1 year [28]. They also suggest collecting blood samples for further translational studies in order to discover the underlying pathophysiological process. 

There have been two published studies so far about critically ill COVID-19 patients followed in post-intensive care outpatient clinics. The first study [29] was a preliminary report about 3-month QOL (assessed by SF-36) in survivors of severe ARDS due to COVID-19 from a single center in France. Nineteen patients were followed in a dedicated ICU follow-up service. They showed impaired scores in all components of the SF-36. The other study [30] was similarly carried out in a single-center critical care recovery clinic from the United States. Forty-five patients were remotely assessed with telephone with patient-reported outcome measures in terms of PICS 1 month after hospital discharge. Patients were asked to answer questionnaires for physical, cognitive, and psychiatric domains for PICS diagnosis. They revealed that 91%, 53.6%, and 4.9% of the patients had dysfunction in at least one domain, two domains, and in all three domains of PICS, respectively. 

## 4. I-POINT protocol

**Figure 2:**
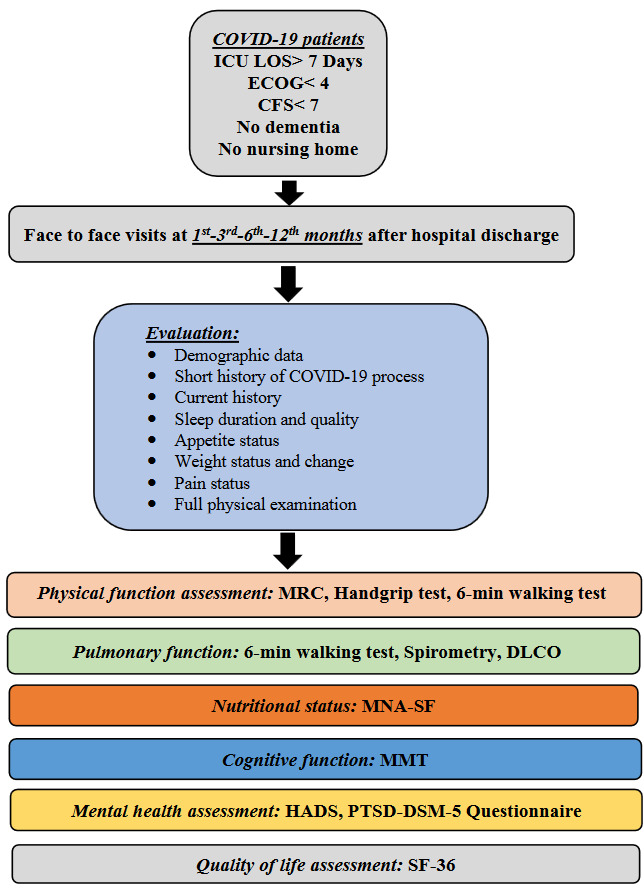
I-POINT protocol. ICU LOS: intensive care unit length of stay, ECOG: Eastern Cooperative Oncology Group, CFS: Clinical Frailty Scale, MRC: Medical Research Council, DLCO: Diffusion capacity of carbon monoxide, MNA-SF: Mini Nutritional Assessment Short Form, MMT: Mini-mental test, HADS: Hospital Anxiety and Depression Scale, PTSD-DSM: Post-Traumatic Stress Disorder-Diagnostic and Statistical Manual of Mental Disorders, SF: Short Form.

The implementation process of the Post-Intensive Care Outpatient Clinic in Hacettepe University Department of Internal Medicine, Division of Intensive Care Medicine was planned at the end of 2019 and regular patient follow-up was started in April 2020 only after the 1st month of the pandemic. A separate room in the department was reserved. One ICU fellow in training and consultant were in charge for those patients. Critically ill COVID-19 patients hospitalized in our ICU for more than 7 days, with Eastern Cooperative Oncology Group (ECOG) [31] performance score below 4, Clinical Frailty Scale (CFS ©) [32] below 7, not staying in nursing homes and without dementia were invited to the POINT clinic by appointments at regular intervals. Control visits were planned for the patients at the 1st, 3rd, 6th, and 12th months starting from the hospital discharge (Figure 2) and whenever needed.

The demographic data of the patients, ICU and hospital admission and discharge dates were recorded. History including sleep duration and quality, pain, and appetite and feeding status of the patients were questioned. Current height and weight measurements, prehospital and discharge weights were recorded and a full physical examination was performed. Level of pain was determined with visual analogue scale (VAS), and the level of dyspnea with Borg scale [33].

### 4.1. Assessment of physical and pulmonary function status

Muscle function is evaluated by our intensive care physiotherapist at ICU discharge and during outpatient control visits. Medical Research Council (MRC) tool is utilized to evaluate strength of 6 muscle groups bilaterally which is scored from 0 to 60 (maximum strength). A score of <48 is accepted as “ICU-acquired weakness” [34]. In addition, handgrip test with a hand dynamometer is applied during outpatient controls. A test result of <14 kg in women and <28 kg in men is considered as muscle weakness as defined previously in the Turkish population [35]. 

A standardized 6MWT is done on a 20-m hallway marked at 1-m intervals within the department to evaluate functional capacity and pulmonary function during each outpatient visit. Vital signs, levels of dyspnea, and fatigue are recorded with Borg scale before and after the test. 6MWT is continued with a pulse oximeter and the longest walked distance is recorded. The expected distance according to the patient’s age is calculated according to the formula determined by sex [6 min walking distance for male patients = (7.57× ´ height in cm) – (5.02× ´ age) – (1.76× ´ weight in kg) – 309, for female patients = (2.11× ´ height in cm) – (2.29× ´ weight in kg) – (5.78× ´ age) + 667] [36]. Percentage of the completed distance is calculated according to expected distance [37]. Spirometry and DLCO tests are requested when needed and from those who could go to the pulmonary function laboratory. 

### 4.2. Evaluation of nutritional status

There are several nutritional assessment tools for hospitalized patients endorsed by international societies such as Nutritional Risk Screening (NRS)-2002 and Malnutrition Universal Screening Tool (MUST) [38]. The short form of the Mini Nutritional Assessment (MNA-SF) questionnaire is being used as it is practical [39]. Even if MNA-SF was validated for elderly population, the applicability of MNA-SF was also shown in nongeriatric patients [40]. In the short form, presence of weight loss, functional limitation, psychological stress in the last 3 months, and dementia are questioned. Total score ranges from 0 to 14, <8 as presence of malnutrition and 8–11 as having risk of malnutrition. Prehospital nutritional status, as well as nutritional status at discharge and control visits, is recorded. 

### 4.3. Assessment of cognitive function

Among the neuropsychiatric examination methods, mini-mental test (MMT) [41], which was established as a cognitive assessment tool that takes shorter time to apply, was selected as the test used to quantitatively evaluate cognitive performance like Montreal Cognitive Assessment (MoCA) tool which contains too many questions and take too much time in practice [42]. MMT test includes 30 directions and questions with a score ranging from 0 to 30 points. Scores of >23 are accepted as normal, 19–23 points as mild, 10–18 points as moderate, and those who scored <10 as severe cognitive impairment. Level of orientation as knowing daily life information, their short memory states, ability to understand and apply what is said and what they read were evaluated. 

### 4.4. Mental health assessment

For this purpose, Hospital Anxiety and Depression Scale (HADS) [43] and Post-traumatic Stress Disorder-Diagnostic and Statistical Manual of Mental Disorders-5 (PTSD) (DSM-5) Questionnaire are used. In both scales, questions are asked to determine the situation reflecting the last month. In the HADS questionnaire, there are eleven questions. The answers given to half of the questions seek anxiety; the other half is for depression evaluation. HADS has a total score with range of 0–21. For both anxiety and depression, <8 points are accepted as normal, 8–10 points as borderline, and >10 points as abnormal. A score >48 out of a maximum of 80 points from the PTSD questionnaire is accepted as PTSD as described previously in the Turkish population [44].

### 4.5. Quality of life assessment

There are two accepted health-related quality of life measurement tools including European quality of life questionnaire-5 dimensions (EQ-5D)^3^ and the SF-36 [45], both of them have been validated in critically ill patients. The latter which evaluates the QOL within the last month is used in our patients during outpatient controls. SF-36 consists of 8 parts and 36 questions from 0 to 100 points which are grouped into two main components named as Physical Component Score (PCS) and Mental Component Score (MCS). PCS and MCS are calculated as described elsewhere [45]. A score of <50 points is considered a poor QOL for PCS and MCS. The domains of the PCS are physical functioning, role limitation due to physical health, body pain and general health, while the domains that make up the MCS are vitality, social functioning, role limitation due to emotional problems and mental health.

## 5. Conclusion

Health is defined as not only the absence of illness and/or disability, but also a state of complete physical, mental, and social well-being as accepted by the World Health Organization (WHO) [46]. Therefore, follow-up of the critically ill patients who survived COVID-19 which is thought to have long-term effects on patients, should be systematically performed from this point of view by all aspects. Considering that PICS do not even have an International Classification of Disease Diagnostic (ICD) Code yet, it has been suggested that an ICD code should be created for PICS within the era of COVID-19 pandemic [47].

^3^EuroQol Research Foundation. EQ-5D-3L User Guide, 2018 [online]. Website: https://euroqol.org/publications/user-guides [accessed December 2018].
